# From Prophylactic Screening to Definitive Treatment: A Case Report of Breast Cancer in a Young Woman

**DOI:** 10.7759/cureus.101722

**Published:** 2026-01-17

**Authors:** Sandra Czyz, Wiktoria Marszal, Rafal Marszal

**Affiliations:** 1 Medical Education, Academy of Silesia, Katowice, POL; 2 Psychiatry, Wojewódzki Szpital Zespolony im. Ludwika Perzyny w Kalisz, Kalisz, POL

**Keywords:** a high-risk patient, breast reconstruction surgery, cancer genetic testing, invasive carcinoma of no special type, left breast cancer, mastectomy

## Abstract

This article presents the case of a 39-year-old woman with a positive family history of breast cancer (her family member had left breast cancer, cT4N2M0, non-luminal human epidermal growth factor receptor 2 (HER2)-positive). During routine prophylactic ultrasonographic examination, a breast mass was detected, which, on further diagnostics, turned out to be an invasive ductal carcinoma of the left breast. The stage of the disease was determined as pT1cN1mic(sn), Nottingham Histologic Grade 3 (NHG 3).

The patient was subjected to surgical treatment, which involved left mastectomy with simultaneous removal of the axillary sentinel lymph node. Subsequently, adjuvant chemotherapy was administered, including a "dose-dense" regimen with the use of doxorubicin and cyclophosphamide, followed by treatment with paclitaxel. During chemotherapy, complications related to the implantation of a vascular port occurred, which resulted in its removal before the completion of treatment.

After the completion of chemotherapy, breast reconstruction was performed. During the entire treatment process, extended genetic diagnostics were performed, which did not demonstrate the presence of pathogenic gene mutations. The treatment was completed, and the patient remains under continuous care of the oncology outpatient clinic.

## Introduction

The breasts of both women and men are composed of adipose tissue. In women, the lobes and lobules of the breast are connected with milk ducts. Additionally, numerous nerves as well as blood and lymphatic vessels are present in the breast [1].

Breast cancer constitutes approximately one-third of all malignancies in women and is the most frequently occurring cancer in this population worldwide. The incidence of this disease continues to increase, and the risk of its occurrence rises with age. Countries with higher income are characterized by lower mortality, which is associated with increasingly effective methods of early diagnosis and treatment. One of the basic screening methods that improve early diagnosis of breast cancer is mammography. The main risk factors for morbidity are genetic, environmental, and lifestyle related [1-3].

In the majority of cases, breast cancer takes the form of adenocarcinoma. These neoplasms are divided into non-invasive and invasive types. Non-invasive cancer is limited to the lobule or duct in which it arises; however, it may transform into an invasive form. In invasive cancer, malignant cells spread beyond the lobules and milk ducts, penetrating through the lymphatic system or the systemic circulation. Invasive cancer is the most frequently occurring type of breast cancer in women. In the case of distant metastases, the neoplasm is defined as metastatic cancer, with metastases most commonly occurring in the brain, bones, lungs, and liver [1,3].

The assessment of disease advancement and selection of appropriate treatment are based on the TNM (tumor-node-metastasis) classification [4,5]. This system is used worldwide, which enables effective communication between physicians and medical centers. The name of the TNM classification refers to tumor characteristics (size), regional lymph nodes (number, localization, size), and the presence or absence of distant metastases [5].

The basic methods of breast cancer treatment are surgery, chemotherapy, radiotherapy, hormone therapy, and targeted therapy. In patients with an increased risk of disease development, bilateral prophylactic mastectomy may be performed [1,2]. Preoperative chemotherapy enables a reduction of tumor size and facilitates the performance of surgical treatment [4].

## Case presentation

This case report describes a 39-year-old woman with a positive family history of breast cancer. The patient’s mother suffered from breast cancer; therefore, the patient prophylactically underwent breast imaging examinations. Additionally, she maintained a healthy diet, exercised regularly, and completely abstained from alcohol consumption.

The first mammographic examination was performed on March 9, 2018, when the patient was 39 years old. In the image of both breasts, a glandular-fatty structure and bilateral mastopathic changes were identified. Skin thickening, clusters of microcalcifications, or mass-like shadows were not present. The patient was referred for ultrasonographic examination.

**Figure 1:**
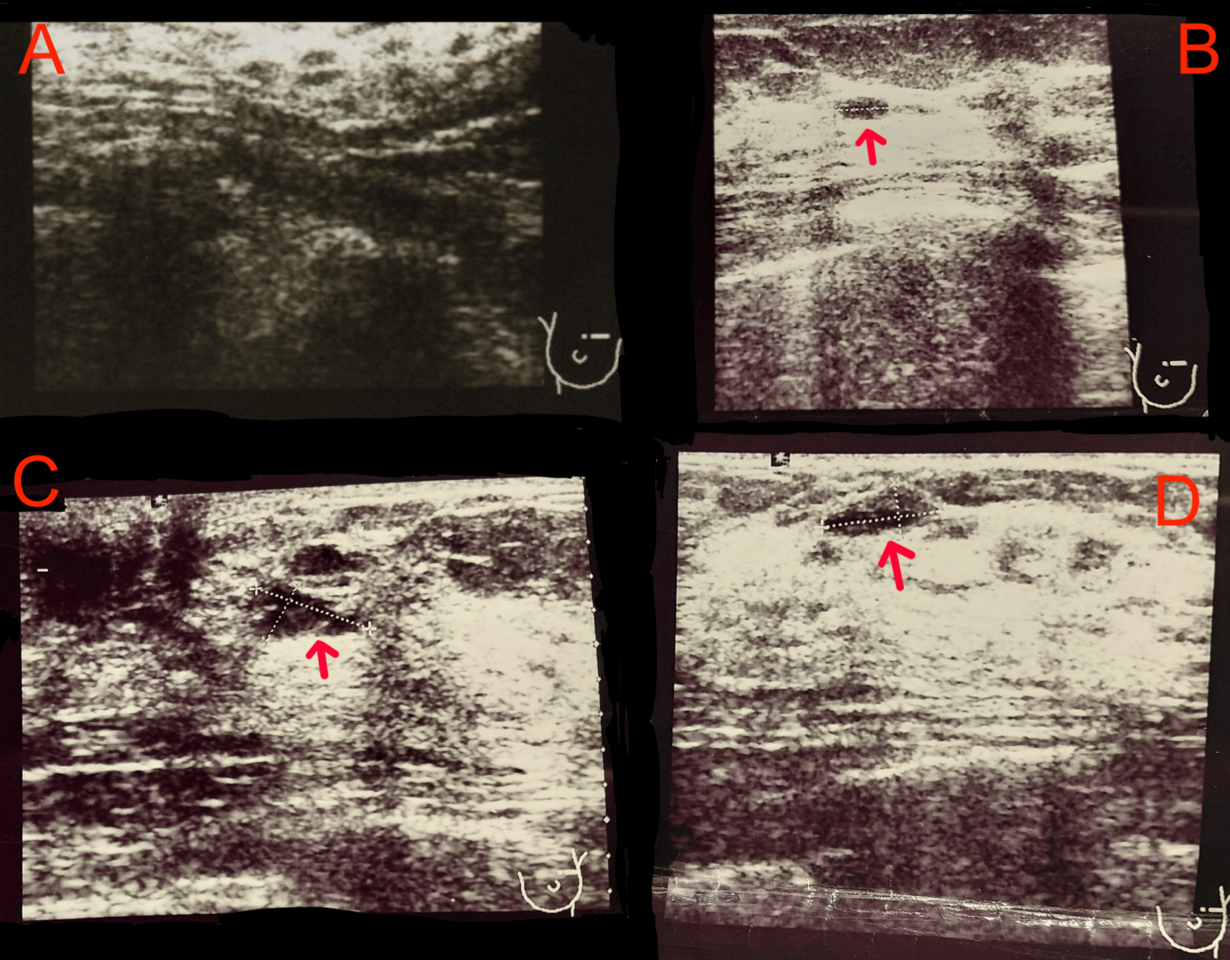
Breast ultrasound examination on March 13, 2018 A: Breast with predominantly glandular-adipose composition; B: Cyst measuring 5 mm in diameter (red arrow); C: Cluster of milk ducts measuring 7×4 mm (red arrow); D: Lesion of mixed echogenicity measuring 9×4 mm, suggestive of a mastopathic cluster (red arrow).

On March 13, 2018, ultrasonography (US) of the breasts and axillary fossae was performed. The image of both breasts corresponded to a glandular-fatty structure with bilateral mastopathic changes of moderate severity and the presence of cysts with a diameter of 5-6 mm. In the left breast, at the two o’clock position, an area of glandular tissue with mixed echogenicity, measuring 9×4 mm, was visualized, suggesting a mastopathic cluster. Additionally, in the left breast, periareolar at the three o’clock position, a cluster of milk ducts measuring 7×4 mm was identified. The axillary fossae showed no visible pathologies (Figure 1).

Due to the burdensome family history, regular follow-up examinations were recommended for the patient. In subsequent years (2019 and 2020), annual breast ultrasonography examinations did not demonstrate the progression of the lesions compared with the examination performed in 2018.

**Figure 2:**
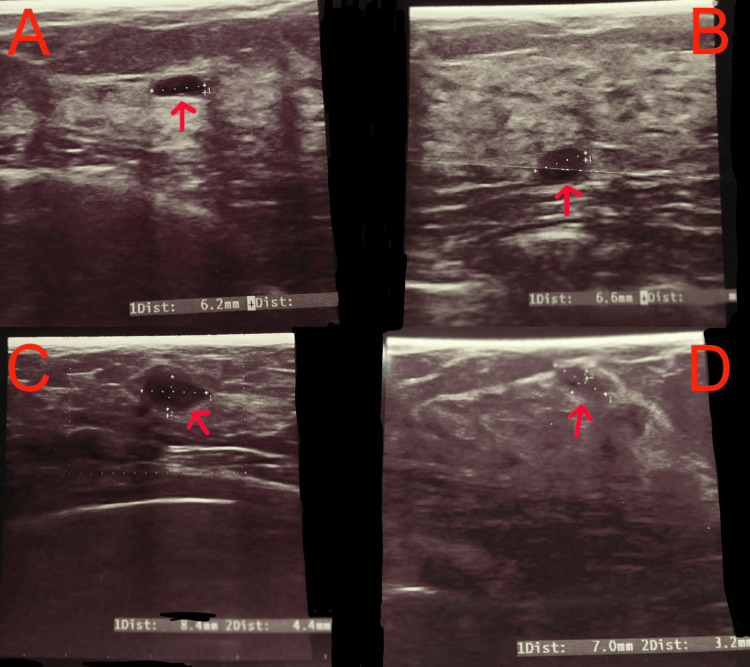
Breast ultrasound examination on November 23, 2020 A: Cyst measuring 6.2 mm in diameter (red arrow); B: Cyst measuring 6.6 mm in diameter (red arrow); C: Cyst measuring 8.4 mm in diameter (red arrow); D: Area of reduced echogenicity measuring 7×3 mm (red arrow), suggestive of a dysplastic area.

In November 2020, the patient independently palpated a lump in the left breast. Ultrasonographic examination performed on November 23, 2020, did not reveal significant differences compared with previous results (Figure 2).

**Figure 3:**
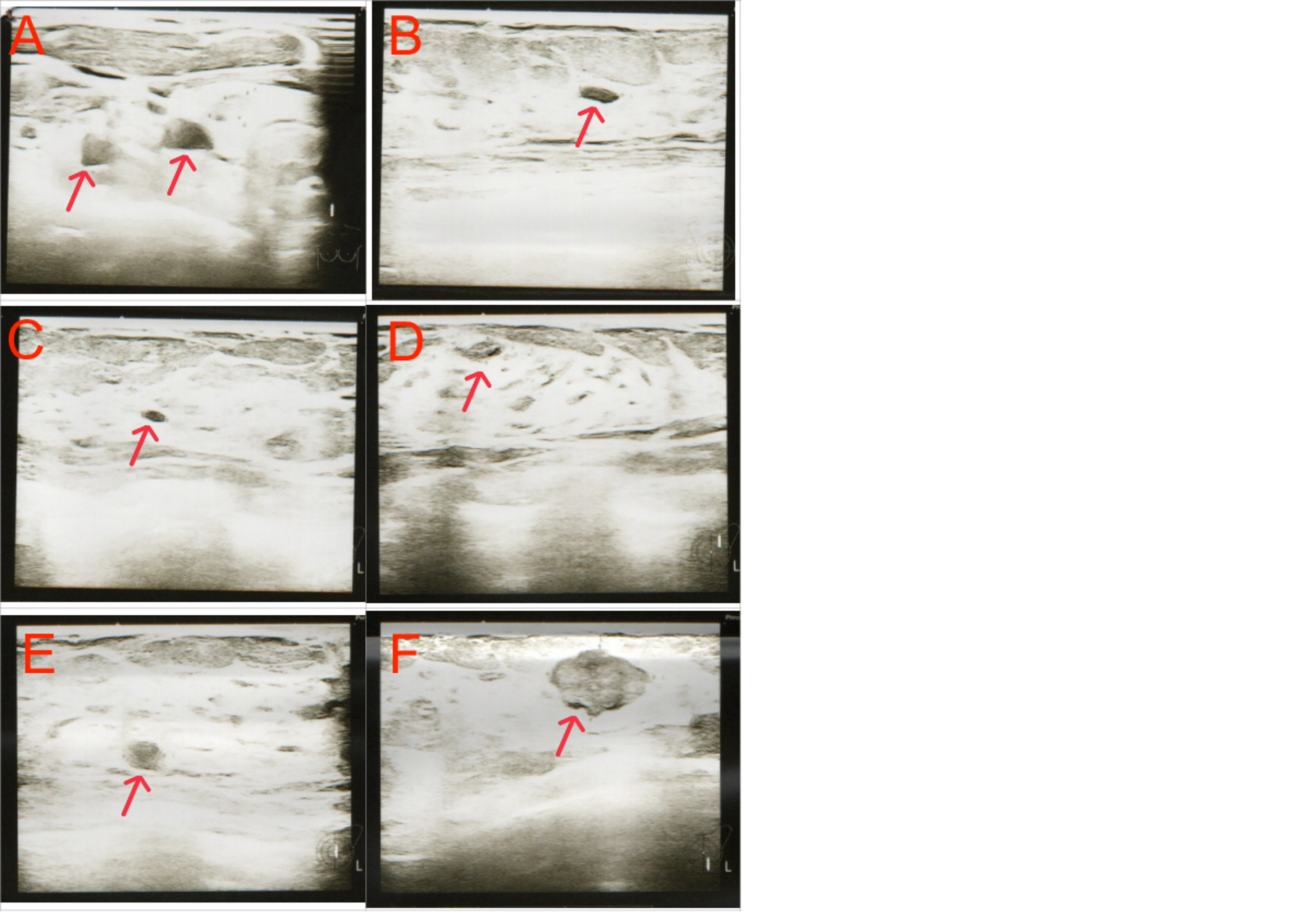
Breast ultrasound examination on March 17, 2021 A: Cysts measuring 7.3 mm and 4.1 mm in diameter; B: Cyst measuring 7 mm in diameter; C: Cyst measuring 4.3 mm in diameter; D:  Hypoechoic transformation measuring 8×4 mm, suggestive of a milk duct filled with dense content; E: Cyst measuring 6.8 mm in diameter; F: Hypoechoic lesion measuring 18×13×13 mm with increased vascular flow.

Due to the patient’s increasing anxiety and the progressively more palpable lump, a follow-up ultrasonographic examination was performed on March 17, 2021. In the left breast, at the five o’clock position, a hypoechoic lesion measuring 18×13×13 mm with increased vascular flow was identified. The axillary, supraclavicular, and infraclavicular lymph nodes showed no pathological features (Figure 3).

The patient was urgently referred to the oncology outpatient clinic. On March 18, 2021, a core needle biopsy of the lesion in the left breast was performed. Histopathological examination revealed invasive breast carcinoma of the NST (no special type) type, NHG 3 (Nottingham Histologic Grade).

After obtaining the biopsy result, the patient was referred for molecular testing to assess the amplification of HER2 (human epidermal growth factor receptor 2) gene. The examination performed using the fluorescence In situ hybridization (FISH) method demonstrated no amplification of the HER2 gene. Subsequently, the patient was recommended for urgent surgical treatment.

On March 29, 2021, the patient was admitted to the clinical department of breast tumors and reconstructive surgery for an urgent breast surgery procedure. On the day of admission, vital parameters were normal (body temperature 36.4°C, blood pressure 123/69 mmHg, heart rate 80/min), and pain intensity was assessed as 0 on the Visual Analogue Scale (VAS). On the following day, a simple amputation of the left breast with identification and removal of the left axillary sentinel lymph node was performed. During the procedure, a breast tissue expander (Polytech, Dieburg, Germany) was implanted. The patient was discharged from the hospital on March 8, 2021, in good general condition, with a recommendation to present for follow-up after two to four days.

Postoperative material was submitted for histopathological examination, the result of which was available on March 30, 2021. The examination confirmed the presence of invasive ductal carcinoma of the left breast. The stage of the disease was determined as pT1cN1mic(sn), which means: pT1c - pathological stage of the primary tumor with the greatest dimension >1 cm and ≤2 cm, N1mic - presence of micrometastases in lymph nodes with a diameter ≤2 mm, and (sn) - involvement of the sentinel node. Macroscopically, the left breast weighed 512 g and measured 17.5×17×2.5 cm, without the skin. In the lower outer quadrant, a tumor measuring 2×1.5×1.5 cm was present, with a margin of 1 cm from the fascia and more than 1 cm from the remaining resection lines. Mastopathic changes were also present.

Microscopic examination revealed invasive breast carcinoma of the NST type, NHG 3, with surgical margins free of neoplastic infiltration. No features of vascular or neural invasion were identified. Apart from the neoplastic tumor, mastopathic changes with adenosis were present. Histopathological examination of the left axillary sentinel lymph node (dimensions 2×1.5×1 cm) revealed the presence of a micrometastasis of carcinoma measuring 0.15 cm, without extracapsular extension.

After the surgical procedure, adjuvant chemotherapy was recommended for the patient. The first cycle of chemotherapy in the "dose-dense” regimen with the use of doxorubicin and cyclophosphamide was administered on May 17, 2021. The "dose-dense” regimen is a chemotherapy approach consisting of administration of standard doses of cytotoxic agents at shorter time intervals than in classical protocols [6].

Due to the planned multiple cycles of chemotherapy, the patient was referred for implantation of a vascular access port. On May 26, 2021, she was admitted to the surgical department; however, due to leukopenia identified in laboratory tests, she was disqualified from the procedure (Table 1).

**Table 1:** Blood laboratory tests on May 26, 2021 β-HCG: Beta Human Chorionic Gonadotropin, HGB: Hemoglobin, HCT: Hematocrit, RBC: Red Blood Cells, WBC: White Blood Cells, PLT: Platelets, MCV: Mean Corpuscular Volume, MCH: Mean Corpuscular Hemoglobin, MCHC: Mean Corpuscular Hemoglobin Concentration, RDW-CV: Red Cell Distribution Width – Coefficient of Variation, P-LCR: Platelet Large Cell Ratio, NRBC: Nucleated Red Blood Cells, eGFR: Estimated Glomerular Filtration Rate, ALT: Alanine Aminotransferase, AST: Aspartate Aminotransferase, IG: Immature Granulocytes

Tested parameter	Result	Reference values
β-HCG	<0.200	0-5.3 mIU/mL for premenopausal women; 0–8.3 mIU/mL for postmenopausal women
SARS-CoV Test	Negative	
HGB	11.6 g/dl	12.0-16.0 g/dl
HCT	35.0 %	37.0-47.0%
RBC	4.13 ×10¹²/L	4.00-5.00×10¹²/L
WBC	1.64 ×10⁹/L	4.00-10.00×10⁹/L
PLT	194 ×10⁹/L	130-350×10⁹/L
MCV	84.7 fl	84.0-94.0 fl
MCH	28.1 pg	27.0-34.0 pg
MCHC	33.1 g/dl	31.0-37.0 g/dl
RDW-CV	13.0 %	11.5-14.5%
P-LCR	33.7%	19.5-43.8%
NRBC	0.000×10⁹/L	0.000-0.015 ×10⁹/L
NRBC %	0.000/100 WBC	0.000-0.030 WBC
Creatinine	72.10 µmol/l	45.00-84.00 µmol/l
eGFR	76.9 ml/min/1.73 m	
Total Bilirubin	4.10 µmol/l	0.00-21.00 µmol/l
ALT	15.6 U/l	0.0-33.0 U/l
AST	13.2 U/l	0.0-32.0 U/l
Basophils	0.02 ×10⁹/L	0.05-0.15×10⁹/L
Basophils %	1.2%	0.0-2.0%
Neutrophils	0.32 ×10⁹/L	1.60-7.50×10⁹/L
Neutrophils %	19.5%	45.0-75.0%
Eosinophils	0.11 ×10⁹/L	0.10-0.50×10⁹/L
Eosinophils %	6.7%	0.0-7.0%
Lymphocytes	0.98×10⁹/L	1.50-4.50×10⁹/L
Lymphocytes %	59.8%	16.0-45.0 %
Monocytes	0.20×10⁹/L	0.40-0.80×10⁹/L
Monocytes %	12.2%	4.0-10. %
IG	0.01×10⁹/L	0.01-0.04×10⁹/L
IG %	0.60%	0.16-0.62%

On physical examination, the following were found: general condition according to the Zubrod scale 1; skin clean, without pathological eruptions; nutritional status normal; musculoskeletal system within normal limits; head symmetrical, non-tender on percussion; eyeballs properly positioned, pupils round, equal, and reacting properly to light; neck symmetrical, properly mobile, thyroid gland symmetrical, not enlarged; chest properly arched and mobile; over the lung fields bilaterally, physiological vesicular breath sounds present; heart activity regular, 78/min; abdomen soft, non-tender on palpation, without pathological resistance; liver not enlarged, spleen non-palpable; Goldflam’s sign negative bilaterally; urinary system without deviations; breasts - status after simple amputation of the left breast. In the recommendations, antibiotic therapy with clarithromycin (Klabax) at a dose of 500 mg twice daily and steroid therapy were recommended. Additionally, the use of dexamethasone (Pabi Dexamethason) at a dose of 500 µg - two tablets in the morning and one at noon - as well as pantoprazole (Contix) 20 mg twice daily was recommended.

Despite the lack of a vascular access port, on May 31, 2021, the patient received the second cycle of chemotherapy in the „dose-dense” regimen with the use of doxorubicin and cyclophosphamide.

Due to difficulties in obtaining venous access during chemotherapy administration, the patient was again referred for implantation of a vascular access port. On June 9, 2021, she was admitted in an accelerated mode to the surgical ward. Upon admission, laboratory blood tests were performed (Table 2).

**Table 2:** Blood laboratory tests on June 9, 2021 HGB: Hemoglobin, HCT: Hematocrit, RBC: Red Blood Cells, WBC: White Blood Cells, PLT: Platelets, MCV: Mean Corpuscular Volume, MCH: Mean Corpuscular Hemoglobin, MCHC: Mean Corpuscular Hemoglobin Concentration, RDW-CV: Red Cell Distribution Width - Coefficient of Variation, RDW-SD: Red Cell Distribution Width - Standard Deviation, PDW: Platelet Distribution Width, MPV: Mean Platelet Volume, P-LCR: Platelet Large Cell Ratio, PCT: Plateletcrit, INR: International Normalized Ratio, APTT: Activated Partial Thromboplastin Time

Tested parameter	Result	Reference values
HGB	10.6 g/dl	12.0-16.0 g/dl
HCT	32.6%	37.0-47.0%
RBW	3.69×10¹²/L	4.00-5.00×10¹²/L
WBC	3.40×10⁹/L	4.00–10.00×10⁹/L
PLT	275×10⁹/L	130-350×10⁹/L
MCV	88 fl	84.0-94.0 fl
MCH	29 pg	27.0-34.0 pg
MCHC	32.5 g/dl	31.0-37.0 g/dl
RDW-CV	13.9%	11.5-14.5%
RDW-SD	43 fl	36-47 fl
PDW	11.1 fl	10-15 fL
MPV	9.8 fl	7.5-10.5 fl
P-LCR	24.3%	19.5-43.8%
PCT	0.27%	0.14-0.36%
Basophils	0.05×10⁹/L	0.05-0.15 ×10⁹/L
Basophils %	1.5%	0.0–2.0%
Neutrophils	1.93×10⁹/L	1.60-7.50×10⁹/L
Neutrophils %	156.7%	45.0-75.0%
Eosinophils	0.01×10⁹/L	0.10-0.50×10⁹/L
Eosinophils %	0.3%	0.0-7.0%
Lymphocytes	0.86 ×10⁹/L	1.50-4.50×10⁹/L
Lymphocytes %	25.3%	16.0-45.0%
Monocytes	0.55×10⁹/L	0.40-0.80×10⁹/L
Monocytes %	16.2%	4.0-10.0 %
Neutrophil Myelocytes	2%	0%
Neutrophil Metamyelocytes	3%	0%
Band Neutrophils	10%	1-6%
Segmented Neutrophils	44%	50-70%
Basophilic Granulocytes	1%	0-1%
Lymphocytes	25%	20-40%
Monocytes	15%	2-8%
INR	0.95	0.8-1.2
Prothrombin Time	11.3 seconds	11–15 seconds
APTT	24.5 seconds	25–35 seconds
SARS-CoV Test	Negative	

All performed examinations were within normal limits, and therefore implantation of a vascular access port in the right subclavian fossa was carried out. After the procedure, a control chest radiograph was performed. On the chest X-ray in the PA projection, the tip of the vascular port was visualized in the correct position within the projection of the superior vena cava. Additionally, a calcified nodular lesion with a diameter of 6 mm was noted in the middle-upper field of the right lung. The lung fields were free of consolidations and showed no signs of increased interstitial markings; pulmonary circulation was normal. The pleural cavities were free, without evidence of fluid or pneumothorax. The cardiothoracic silhouette remained normal. The patient was discharged from the hospital the following day in good general condition, with a recommendation for prophylactic use of enoxaparin (Clexane) at a dose of 4000 IU/0.4 ml once daily.

The patient received two additional cycles of chemotherapy with doxorubicin and cyclophosphamide, administered on June 14 and June 28, 2021, respectively. Administration of chemotherapy via the vascular port proceeded without complications.

On July 10, 2021, the patient was urgently admitted to the oncological surgery ward due to an abscess at the site of the vascular port implantation. On the same day, the vascular port was removed surgically. During the procedure, purulent material from the postoperative wound was collected for microbiological examination. After 48 hours of incubation, the aerobic culture was negative.The patient was discharged on July 12, 2021, in good general condition, with the surgical wound healing. Laboratory blood tests were performed during hospitalization (Table 3). A follow-up visit to the outpatient clinic was recommended after four days.

**Table 3:** Blood laboratory tests on July 10, 2021 HGB: Hemoglobin, HCT: Hematocrit, RBC: Red Blood Cells, WBC: White Blood Cells, PLT: Platelets, MCV: Mean Corpuscular Volume, MCH: Mean Corpuscular Hemoglobin, MCHC: Mean Corpuscular Hemoglobin Concentration, RDW-CV: Red Cell Distribution Width - Coefficient of Variation, P-LCR: Platelet Large Cell Ratio, NRBC: Nucleated Red Blood Cells, INR: International Normalized Ratio, CRP: C-Reactive Protein

Tested parameter	Result	Reference values
HGB	8.8 g/dl	12.0-16.0 g/dl
HCT	28.1%	37.0-47.0%
RBW	3.07×10¹²/L	4.00-5.00×10¹²/L
WBC	29.00 g/l	4.00-10.00×10⁹/L
PLT	227 g/l	130-350 ×10⁹/L
MCV	91.5 fl	84.0-94.0 fl
MCH	28.7 pg	27.0-34.0 pg
MCHC	31.3 g/dl	31.0-37.0 g/dl
RDW-CV	19.8 %	11.5-14.5%
P-LCR	21.6%	19.5-43.8%
NRBC	0.090 g/l	0.000-0.015×10⁹/L
NRBC %	0.300/100 WBC	0.000-0.030 WBC
INR	1.06	0.8-1.2
Prothrombin Ratio	94.12%	70-120%
C-Reactive Protein	4.78 mg/l	<5 mg/L
Sodium	143 mmol/L	135-145 mmol/L
Potassium	3.63 mmol/L	3.5-5.0 mmol/L

After completion of chemotherapy in the "dose-dense” regimen with doxorubicin and cyclophosphamide, the patient underwent adjuvant treatment consisting of 11 cycles of chemotherapy with paclitaxel at a dose of 175 mg/m² per cycle. Treatment was administered on the following dates: July 19 and 26; August 2, 16, 23, and 30; September 6, 13, 20, and 27; and October 4 and 11, 2021.

During treatment, genetic testing for the BRCA1 gene mutation was performed on July 20, 2021, which did not reveal the presence of the analyzed mutation.

A subsequent genetic test was performed on February 22, 2022, analyzing the CHEK2 and PALB2 genes. In this case as well, no pathogenic mutations were detected.

Due to the negative results of previous genetic tests, the patient was referred for extended genetic diagnostics. On May 6, 2022, venous blood was collected for an oncological panel analyzing 81 genes. No known pathogenic variants associated with increased predisposition to cancer were detected in the analyzed panel. No potentially pathogenic variants were identified in the analyzed exons or adjacent intronic regions, and copy number variation (CNV) analysis revealed no deletions in the tested gene panel.

A control ultrasonographic examination performed on June 1, 2022, showed status post left breast amputation and reconstruction. In the area of the scar, small fibrous band-like changes were visualized; the contours of the expander were smooth, with no signs of leakage, and a trace amount of fluid measuring 2-3 mm was present. The right breast had a glandular-fatty structure without any solid focal lesions. Axillary lymph nodes showed no suspicious features.

On November 29, 2022, the patient was admitted to the clinical department of breast tumors and reconstructive surgery for the exchange of the expander to an implant. Upon admission, vital signs were normal (body temperature 36.5°C, blood pressure 121/74 mmHg, heart rate 64/min). During the procedure, an ERSF 500Q True Fixation (Motiva Implants, New York, TX, USA) breast prosthesis was implanted in the left breast. The operative material, consisting of a fragment of the left breast expander capsule measuring 5.5×2.5×0.5 cm, was submitted for histopathological examination. No features of malignancy were identified. Fibrous capsule fragments were described, with sparse chronic inflammatory infiltrate present.

The patient was discharged home on December 5, 2022, in good general condition, with a recommendation for follow-up at the breast disease outpatient clinic and evaluation of wound healing two to four days after discharge.

**Figure 4:**
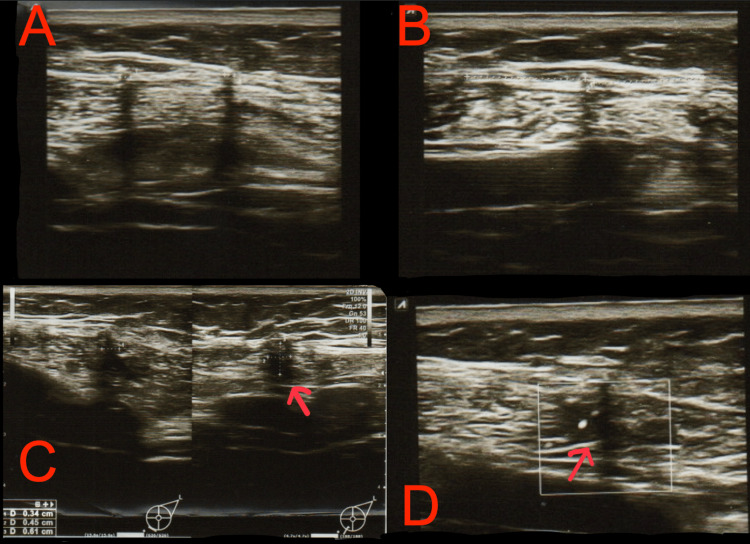
Breast ultrasound examination on January 23, 2023 A: Layered structure of the chest wall tissues after mastectomy with smooth contour of the tissue expander; B: Layered structure of the chest wall tissues after mastectomy with smooth contour of the tissue expander; C: Hypoechoic area measuring 3×5×6 mm with single vascular flows (red arrow); D: Single vascular flows within the 3×5×6 mm lesion (red arrow).

After the procedure, a follow-up ultrasonographic examination was recommended and performed on January 23, 2023 (Figure 4). The examination revealed status post left mastectomy and expander implantation. The contents of the expander were anechoic, and its outer contour was smooth, without signs of damage. In the area indicated by the patient as painful, below the lower pole of the expander at the seven o’clock position, peripherally within the muscle, a hypoechoic area measuring 3×5×6 mm with single vascular flows was visualized. The lesion could correspond to a postoperative change; however, further verification was recommended due to the inability to exclude another etiology. No other focal lesions were identified. The right breast had a mixed glandular-fatty structure. No suspicious lymph nodes were visualized in the axillary, supraclavicular, or infraclavicular regions. An oncological consultation was recommended.

On January 26, 2023, a core needle biopsy of the described lesion in the left breast was performed under ultrasonographic guidance. In the aspirated material, fragments of fibrotic tissue were identified, without evidence of neoplastic cells.

On March 21, 2023, laboratory blood tests were performed to assess the patient’s general health. The detailed results of these tests are presented in Table 4.

**Table 4:** Blood laboratory tests on March 21, 2023 HGB: Hemoglobin, HCT: Hematocrit, RBC: Red Blood Cells, WBC: White Blood Cells, PLT: Platelets, MCV: Mean Corpuscular Volume, MCH: Mean Corpuscular Hemoglobin, MCHC: Mean Corpuscular Hemoglobin Concentration, RDW-CV: Red Cell Distribution Width - Coefficient of Variation, PDW: Platelet Distribution Width, MPV: Mean Platelet Volume, TSH: Thyroid Stimulating Hormone, AIAT: Alanine Aminotransferase (ALT), AspAT: Aspartate Aminotransferase (AST), GFR: Glomerular Filtration Rate

Tested parameter	Result	Reference values
HGB	12.9 g/dL	12.00-15.40 g/dL
HCT	39%	35.50-45.00%
RBW	4.41×10¹²/L	3.90-5.15 ×10¹²/L
WBC	5.74 ×10⁹/L	3.90-10.2×10⁹/L
PLT	289 ×10⁹/L	150-370×10⁹/L
MCV	88.4 fl	80.00-99.00 fl
MCH	29.3 pg	27.00-33.50 pg
MCHC	33.1 g/dl	31.50-36.00 g/dl
RDW-CV	13.7 %	11.7-14.4%
P-LCR	36 %	19.10-46.6%
PDW	14.8 fl	9.8-16.20 fl
MPV	11.5 fl	9.4-12.5 fl
Potassium	4.58 mmol/L	3.5-5.10 mmol/L
THS	2.990 µIU/mL	0.27-4.2 µIU/mL
Iron	102.2 µg/dL	50.00-170.00 µg/dL
AIAT	16.64 U/l	0.00-36.00 U/l
AspAT	20.55 U/l	Up to 32.00 U/l
Rheumatoid Factor (RF)	8.86 lU/mL	Up to 14.00 lU/mL
Glucose	89.68 mg/dl	70–99 mg/dL → normal fasting glucose 100–125 mg/dL → impaired fasting glucose ≥126 mg/dL → diabetes
Creatinine	0.73 mg/dl	0.00-0.90 mg/dl
GFR	86.02 ml/min/1.73 m^2^	From 60.00 ml/min/1.73 m^2^

On the same day, urine tests were also conducted, and the findings are summarized in Table 5. All results from both the blood and urine analyses were within normal reference ranges, showing no deviations from expected values.

**Table 5:** Urine laboratory tests on March 21, 2023 WBC: White blood cells; RBW: Red blood cell distribution width

Tested parameter	Result	Reference values
Clarity	Slightly cloudy	Clear
Color	Yellow	Yellow
Specific Gravity	1.020 g/ml	1.015-1.030 g/ml
pH	5.0	4.5-8.0
Glucose	Absent	Absent
Ketone Bodies	Absent	Absent
Protein	Absent	Absent
Bilirubin	Absent	Absent
Urobilinogen	Within normal limits	0-4 mg/dl
Nitrites	Absent	Absent
WBC	Absent	Absent
RBW	Absent	Absent
Sediment	Squamous epithelial cells: single per field of view. WBC: 2-4 per field of view, Bacteria: fairly numerous per field of view	Squamous epithelial cells: 3-5 per field of view, WBC: 0-3 per field of view, Bacteria: absent or present in trace amounts

On June 1, 2023, follow-up blood tests were carried out to monitor the patient’s ongoing health status. The results of this examination are provided in Table 6. Similar to the previous assessment, all measured parameters remained within normal limits, showing no evidence of any pathological changes.

**Table 6:** Blood laboratory tests on June 1, 2023 HGB: Hemoglobin, HCT – Hematocrit, RBC: Red Blood Cells, WBC: White Blood Cells, PLT: Platelets, MCV: Mean Corpuscular Volume, MCH: Mean Corpuscular Hemoglobin, MCHC: Mean Corpuscular Hemoglobin Concentration, RDW-CV: Red Cell Distribution Width - Coefficient of Variation, P-LCR: Platelet Large Cell Ratio, PDW: Platelet Distribution Width, MPV: Mean Platelet Volume, GFR: Glomerular Filtration Rate

Tested parameter	Result	Reference values
HGB	13.7 g/dL	12.00-15.40 g/dL
HCT	40.7%	35.50-45.00 %
RBW	4.62×10¹²/L	3.90-5.15×10¹²/L
WBC	5.17×10⁹/L	3.90-10.2 ×10⁹/L
PLT	248×10⁹/L	150-370 ×10⁹/L
MCV	88.1 fl	80.00-99.00 fl
MCH	29.7 pg	27.00-33.50 pg
MCHC	33.7 g/dl	31.50-36.00 g/dl
RDW-CV	13.2 %	11.7-14.4%
P-LCR	40.1%	19.10-46.6%
PDW	15.7 fl	9.8-16.20 fl
MPV	12.1 fl	9.4-12.5 fl
Creatinine	0.75 mg/gl	00.00-0.90 mg/gl
GFR	84.37 ml/min/1.73 m^2^	From 60.00 ml/min/1.73 m^2^
Iron	99.02 µg/dL	50.00-170.00 µg/dL

During this period, a prophylactic mammography of the breasts was also performed to ascertain the status post simple left breast amputation. The right breast demonstrated predominance of glandular tissue with scattered fibrous changes. No skin thickening, clusters of microcalcifications, or mass like shadows were observed.

The patient was recommended to receive zoledronic acid approximately every six months. The first dose was administered on August 11, 2023, followed by doses on February 26, 2024, August 29, 2024, and February 17 and July 31, 2025. After each infusion, the patient reported adverse effects including nausea, vomiting, general weakness, and drowsiness, lasting approximately five days.

On May 5, 2025, a follow-up mammography was performed, which again showed no skin thickening, clusters of microcalcifications, or mass-like shadows.

Currently, the patient remains under continuous care at the oncology outpatient clinic, with a recommendation for follow-up every six months, including ultrasonographic examination of the breasts.

## Discussion

This case report describes the clinical course of breast cancer in a young patient with a positive family history of malignancy. The malignancy was detected at an early stage due to the patient’s participation in regular prophylactic mammographic and ultrasonographic screening. Early detection in young women at increased familial risk is widely recognized as a key factor in improving prognosis; however, this case demonstrates that early-stage diagnosis does not necessarily preclude aggressive tumor biology [7].

Except for a palpable lump, the patient presented no additional clinical symptoms, yet histopathological examination revealed a high-grade carcinoma. Previous studies have shown that breast cancer in younger patients is more frequently associated with high-grade tumors, increased proliferation rates, and less favorable biological features compared with cancers diagnosed in older women [8-10]. This observation is consistent with the findings in the present case and highlights the potential for aggressive disease even in the context of proactive surveillance.

The primary management consisted of surgical treatment with a simple left mastectomy and sentinel lymph node biopsy, followed by adjuvant systemic therapy. The patient received "dose-dense” chemotherapy with doxorubicin and cyclophosphamide, followed by paclitaxel. This treatment approach is consistent with current guidelines for high-risk, early-stage breast cancer and has been shown in the literature to reduce recurrence rates and improve disease-free survival in patients with unfavorable pathological features [11].

Given the patient’s young age and positive family history, a hereditary breast cancer syndrome was strongly suspected. However, comprehensive genetic testing, including BRCA1, CHEK2, PALB2, and an extended multigene panel, did not identify any pathogenic variants. This finding aligns with published data indicating that a substantial proportion of early-onset breast cancers occur in patients without detectable germline mutations. These cases underscore the limitations of current genetic testing and emphasize the continued importance of clinical risk assessment, family history, and long-term surveillance independent of genetic results [12].

During follow-up, no evidence of local recurrence or distant metastasis was observed, indicating a favorable response to the applied multimodal treatment. This case highlights the importance of individualized management strategies, combining early detection, appropriate surgical intervention, and tailored adjuvant therapy. Furthermore, it reinforces the need for ongoing vigilance in young patients with breast cancer, even in the absence of identifiable genetic predisposition.

## Conclusions

The described case confirms that early diagnosis of even highly malignant breast cancer significantly increases the chances of cure. In young patients, prompt initiation of appropriate treatment particularly improves prognosis. The chemotherapy administered to the patient in two regimens proved to be an effective adjuvant therapy following surgery. The use of a vascular access port facilitated the administration of long-term chemotherapy but was also associated with a clinically significant complication, emphasizing that such devices, while beneficial, carry inherent risks. Overall, this case supports the role of vigilant screening and individualized clinical decision-making rather than broad generalization of therapeutic strategies.
